# CO-PRESCRIPTION OF OPIOIDS AND BENZODIAZEPINES AND 30 DAY HOSPITAL RETURNS IN OLDER ADULTS

**DOI:** 10.1093/geroni/igad104.2800

**Published:** 2023-12-21

**Authors:** JuYoung Park, Gabriella Engstrom, Joseph Ouslander

**Affiliations:** Florida Atlantic University, Boca Raton, Florida, United States; Florida Atlantic University, Boca Raton, Florida, United States; Florida Atlantic University, Boca Raton, Florida, United States

## Abstract

With the current opioid epidemic, greater attention has been focused on polypharmacy, in particular use of benzodiazepine (BZD). This study compared prescription of opioids or BZD, co-prescription of both, and prescription of neither at discharge with returns to the hospital within 30 days of discharge of older patients (75+ years). This is a secondary analysis of a quality improvement database developed during implementation of a care transition intervention for hospitalized patients age 75+. The database includes all non-ICU admissions over a 2-year period (24,784 admission cases). Returns to the hospital included Emergency Department visits, inpatient hospital stays, and observation admissions. Among the 24,262 discharges that had medication data, 1,594 (6.6%) were prescribed opioids only, 3,594 (15.0%) BZD only, 544 (2 %) opioids and BZD together, and 18,530 (76.4%) neither opioid or BZD. Each of the four prescription groups had a > 20% return rate within 30 days of discharge: 22.1% opioids only, 23.8% BZDs only, 23.7% opioids and BZDs, and 20.2% neither. Opioids were not associated with a significantly higher incidence of hospital returns. The data highlight the importance of educating healthcare providers about appropriate use of BZD and opioids in hospitalized and post-discharge older patients. Individualized care by interdisciplinary team members and support for family caregivers, as well as careful use of opioids for pain that cannot be managed by other modalities, may reduce hospital return rates and their associated morbidity and costs in the older population.

